# Economic evaluation for first-line anti-hypertensive medicines: applications for the Philippines

**DOI:** 10.1186/1478-7547-10-14

**Published:** 2012-12-10

**Authors:** Lester Sam Araneta Geroy

**Affiliations:** 1 Health Policy, Planning and Financing, London School of Economics and Political Science (LSE) and London School of Hygiene and Tropical Medicine (LSHTM), London, UK

**Keywords:** Economic evaluation, First-line anti-hypertensive medicines, Philippines, Literature review, PhilHealth

## Abstract

**Background:**

Medicines to control hypertension, a leading cause of morbidity and mortality, are a major component of health expenditures in the Philippines. This study aims to review economic studies for first line anti-hypertensive medical treatment without co-morbidities; and discuss practical, informational and policy implications on the use of economic evaluation in the Philippines.

**Methods:**

A systematic literature review was performed using the following databases: MEDLINE, EMBASE, BIOSIS, PubMed, The Cochrane Library, Health Economics Evaluations Database (HEED) and the Centre for Reviews and Dissemination – NHS NICE. Six existing economic analytical frameworks were reviewed and one framework for critical appraisal was developed.

**Results:**

Out of 1336 searched articles, 12 fulfilled the inclusion criteria. The studies were summarized according to their background characteristics (year, journal, intervention and comparators, objective/study question, target audience, economic study type, study population, setting and country and source of funding/conflict of interest) and technical characteristics (perspective, time horizon, methodology/modeling, search strategy for parameters, costs, effectiveness measures, discounting, assumptions and biases, results, cost-effectiveness ratio, endpoints, sensitivity analysis, generalizability, strengths and limitations, conclusions, implications and feasibility and recommendations). The studies represented different countries, perspectives and stakeholders.

**Conclusions:**

Diuretics were the most cost-effective drug class for first-line treatment of hypertension without co-morbidities. Although the Philippine Health Insurance Corporation may apply the recommendations given in previous studies (i.e. to subsidize diuretics, ACE inhibitors and calcium channel blockers), it is uncertain how much public funding is justified. There is an information gap on clinical data (transition probabilities, relative risks and risk reduction) and utility values on hypertension and related diseases from middle- and low- income countries. Considering the national relevance of the disease, a study on the costs of hypertension in the Philippines including in-patient, out-patient, out-of-pocket, local government and national government expenditure must be made. Economic evaluation may be incorporated in health technology assessment, planning, proposal development, research, prioritization and evaluation of health programmes. The approaches will vary depending on the policy questions. The information gap calls for building strong economic evaluative capacity in growing economies.

## Background

Hypertension is a leading cause of mortality and morbidity among chronic diseases in the Philippines [1,2]. Medical costs for hypertension and related morbidities comprise one of the largest costs in reimbursements by the Philippine Health Insurance Corporation (PhilHealth). The Philippine Government, through PhilHealth, is planning to subsidize out-patient pharmaceuticals for relevant diseases and hypertension treatment is one of the target drugs in this out-patient benefit package. Currently, only in-patient medicines are subsidized, albeit at a small proportion of 20% [3]. In this study, I will investigate economic evidence on first-line hypertension treatment worldwide and draw out lessons learned and recommendations for the Philippine context. I will also explore the use of economic evaluation within the broader scope of health technology assessment as a policy tool in the Philippine context.

Hypertension treatment guidelines developed recently have used five main classes of antihypertensive medicine – angiotensin converting enzyme (ACE) inhibitors (ACEIs), angiotensin II receptor blockers (ARBs, also termed angiotensin II receptor antagonists in other sources), beta-blockers (BBs), calcium channel blockers (CCBs, also termed calcium antagonists in other sources) and diuretics. Drugs within each class have basically comparable effects [4,5]. The Philippines Hypertension Guidelines, as published by PhilHealth, recommends that in hypertension stage I without compelling indication, thiazide diuretics are the primary drugs of choice. When BP remains uncontrolled, other classes or combination of drug classes may be used [6]. PhilHealth has outlined compelling indications for different drug classes. Actual use in the Philippines however is different. A recent study showed that based on PhilHealth reimbursements, calcium antagonists, angiotensin II antagonists, beta blockers and ACE inhibitors were used far more than diuretics which only accounted for only 1% [3]. This data represented the use of medicines among hospitalized patients majority of which have co-morbidities. There is no published data in the Philippines on medicines used among out-patients or among patients without co-morbidities.

Hypertension and cardiovascular diseases incur high costs both for patients and their families as well as for society. In developing countries, a great proportion of direct medical costs may fall on individuals in the form of out-of-pocket payments. Hypertension costs are not just related to the disease *per se* but include the costs of future complications. In fact, costs associated with cardiovascular and renal diseases may even be greater because of more expensive medicines, treatment modalities and the complexity required in medical care [7]. The high cost of complications is a good rationale why national governments should invest on hypertension treatment in order to gain savings from long-term care.

A study on claims and reimbursements revealed that PhilHealth has been spending more than PHP 2.1 billion (US$ 49.3 million) per year (2003 to 2005) for 167,666 hospitalizations of patients for hypertension treatment and related conditions. PhilHealth reimbursed a total of 444,628 hypertension-related diagnoses. Given that the average hypertension-related hospitalization bill was PHP 23,210.07 (US$ 544), avoiding expenditures for one hospitalization could pay for one year of anti-hypertensive medicine treatment for three patients. Hospitalizations for heart, renal and other complications of hypertension accounted for 75% of the total costs. Although the study concluded that covering out-patient antihypertensive medicines could actually bring future savings, evidence through economic evaluation is yet to be provided [3].

The costs of medicines in the Philippines are high by international standards and in comparison with both developed and developing countries [8]. Expensive medicines are a challenge especially to the 14% poorest who live on less than $1 per day and the 48% who live on less than $2 per day [3]. About 45-70% of the total costs of medicines are paid out of pocket [9,10] [Unpublished observations, Soe N, WHO Representative in the Philippines, 2010; Unpublished observations, So RL, Philippines Department of Health, 2010].

Policies over the production, sale and use of pharmaceuticals have been developed over the past 20 years. These included regulations on cheaper medicines, intellectual property code, generics and pharmacies. These policies cover the promotion of generics, parallel importation, intellectual property, compulsory licensing, price monitoring and regulation, quality, packaging and sale, prescribing and herbal medicine development [11]. The government has introduced thousands of non-profit community medicines outlets (*Botika ng Barangay*) but their impact on access and reduction of costs remains to be seen [2].

The Philippines has also been publishing the Philippine National Drug Formulary which forms a positive list for PhilHealth reimbursement. Drugs are assessed in terms of safety, efficacy and affordability. PhilHealth has also created the Health Technology Assessment Unit which develops/promotes the use of clinical practice guidelines by assessing the effectiveness, safety and cost-effectiveness of new drugs in comparison with standard treatment and develops recommendations on the indications for their use [6,12]. However, except for tuberculosis, no full economic analysis for hypertension or other diseases has been published. Only cost-benefit analyses for hypertension and diabetes have been published.

Several stakeholders impact the costs and enhance access to medicines [Unpublished observations So N, 2010]. The Philippines Department of Health (DOH) is responsible for national policies that regulate management and access to drugs. The Food and Drug Administration (FDA) is responsible for the safety, efficacy and quality of health products and has a key role for the generic market to flourish. Pharmacoeconomic data is required for market authorization and sales permission [12]. PhilHealth contributes to maintaining the low cost of drugs by setting reference prices to reimbursed medicines in the Philippine National Drug Formulary. Because the Philippine health care system is devolved, local governments and public hospitals can also provide free or low-cost (subsidized) medicines. Bulk purchases by these entities could improve competition and bring prices down [8].

### Objectives

The Philippine Government, through PhilHealth, is planning to subsidize out-patient pharmaceuticals for common diseases including hypertension. This study will provide economic evidence on which anti-hypertensive medicine/s should be covered. It will also explore how economic evaluation may be done to aid policy decisions in covering drugs in other diseases as well as public health programmes. This study aims to review economic studies for first line anti-hypertensive medical treatment without co-morbidities; and discuss practical, informational and policy implications on the use of economic evaluation in the Philippines.

## Methodology

The methodology of this study has two phases: the systematic literature search and the critical appraisal of selected studies. I performed a systematic literature review using the following databases: MEDLINE, EMBASE, BIOSIS, PubMed, The Cochrane Library, Health Economics Evaluations Database (HEED) and the Centre for Reviews and Dissemination – UK National Health Service (NHS) National Institute for Health and Clinical Excellence (NICE). Websites of professional associations were searched. The reference lists of the later selected articles were also reviewed for studies that might be included. Key concepts used in the search were “economic evaluation, “hypertension”, “medicines”, “calcium antagonists”, angiotensin II antagonists”, “angiotensin receptor blockers”, “beta blockers”, “ACE inhibitors” and “diuretics”. I used related key words for each concept.

Studies were included if they met the following inclusion criteria: 1) they were full economic evaluations (cost-effectiveness analysis, cost utility-analysis, cost-benefit analysis or cost-minimization analysis with the statement that effectiveness of comparative alternatives were assumed to be equal) and systematic literature reviews; 2) the intervention in question was the treatment of primary hypertension without co-morbidities; 3) they were done in adults; 4) they were published from 1991 onwards (cut off date June 2011); 5) they were published in English or Spanish; and 6) full texts were available for free or accessible through the London School of Hygiene and Tropical Medicine (LSHTM) library and online network.

Clinical studies focusing on efficacy or safety of pharmaceuticals, studies on costing but not full economic evaluations and studies on hypertension with co-morbidities or in populations with disease subgroups were excluded (exclusion criteria). After reviewing the titles and abstracts, the full texts of eligible studies were obtained.

I reviewed six existing economic analytical frameworks and developed one framework that reflected the essential components as well as the objectives of this study [13-18]. The framework used is divided into two main sections – background characteristics and technical characteristics. The same framework can be used for analysis and drawing of recommendations and lessons learned. I appraised the selected articles based on the framework developed and drew common points using Microsoft Excel. Implications for Philippine application and feasibility in terms of the technical aspect of economic evaluation were noted.

Background characteristics included the following: title, author/s, year, journal, intervention and comparators, objective/study question, target audience, economic study type, study population, setting and country; and source of funding/conflict of interest. Technical characteristics included the following: perspective; time horizon; methodology/modelling; search strategy for parameters; costs; effectiveness measures; discounting; assumptions and biases; results, cost-effectiveness ratio, endpoints; sensitivity analysis; generalizability; strengths and limitations; conclusions; implications and feasibility; and recommendations.

## Results

Database search generated 1336 articles. After reviewing the titles and abstracts using the inclusion and exclusion criteria, I selected 40 articles about economic studies surrounding treatment and other approaches to control, prevention and management. Twelve studies matched my criteria of full economic studies or literature review on first-line treatment of primary hypertension without co-morbidities. These were done by the following authors : Alefan et al. [19], Anderson et al. [20], Tran et al. [16], Chen et al. [21], NICE [22], Fretheim et al. [23], Heidenreich et al. [24], Miller et al. [25], Moreira et al. [26], Moreno et al. [27], Plans-Rubio [28] and Theodoratou et al. [29]. It must be noted that Theodoratou’s literature review [29] gathered economic evidence for two groups of hypertension patients – uncomplicated and with co-morbidities. It was one of the three studies that compared different drugs within the same class; hence, it was included.

### Background characteristics

The studies were conducted from 1998 to 2010. Eight were published in international journals while two came from national journals. Two other studies were published by national HTA agencies (Canada and the UK) as part of their national recommendations for clinical practice and reimbursement coverage. Table 1 summarizes the studies in terms of the population, setting and country type of economic evaluation and conclusions.

**Table 1:** Summary of the studies, medicines compared and conclusions

**Title (author, year)**	**Intervention and comparator**	**Study population**	**Setting and country**	**Type of economic study**	**Conclusions**
Cost-effectiveness of antihypertensive treatment in Malaysia (Alefan et al., [19])	Diuretics, ACE inhibitors, prazosin, BB, diuretic and BB combination, CCB, and other combinations	670 hypertensive patients without comorbidities divided into controlled and uncontrolled groups	A polyclinic in Malaysia	Cost-effectiveness analysis	Diuretics were the most cost-effective antihypertensive drugs followed by ACEIs, prazosin, BB, combination of diuretics and BB, CCBs and “other combinations”.
AT1 Receptor Blockers – Cost-effectiveness within the South African Context (Anderson et al. [20])	Candesartan, losartan, valsartan and irbesartan (all ARB)	Values taken from existing internationally published data	South Africa, but all studies derived from existing international published data	Literature review and cost-effectiveness study	Favoured candesartan as the most cost effective regimen among other ARBs
Thiazide diuretics as first-line treatment for hypertension: Meta-analysis and economic evaluation (Tran et al. [16])	Thiazides, CCB, BB, ACEI or ARB and no therapy	For the base case analysis, the cohorts included men and women, 55–65 years old, with baseline SBP of 150 mmHg or 180 mmHg, all were non smokers with no diabetes or LVH, with normal cholesterol and HDL levels	Data came from studies in the US, Sweden, Spain, UK, Canada, Greece, Italy and New Zealand; cost-utility analysis represented patients from Canada.	Systematic literature review and cost-utility analysis	Favoured thiazides as the most cost-effective option
Cost-minimization analysis of diuretic based antihypertensive therapy reducing cardiovascular events in older adults with isolated systolic hypertension (Chen et al., [21])	SHEP-based therapy (diuretic based plus reserpine or atenolol), BB, ACEI, alpha blocker, and CCB	Men and women, 60 years or more, isolated systolic hypertension, classified into four risk groups: low, medium, high and very high; patients randomized to 2365 treatment group and 2371 placebo group	USA	Cost-minimization analysis	Favoured diuretic based therapy even in patients at high risk for developing cardiovascular disease
Hypertension: Management in adults in primary care (NICE [22])	Thiazide diuretics, CCB, BB, ACEI or ARB, and no intervention	Patients in primary care with essential hypertension without pre-existing CVD, HF or diabetes; divided into different cohorts by age, sex and baseline cardiovascular risk, heart failure risk and diabetes risk	Primary care, UK	Cost-utility analysis	Favoured CCB (associated with low risk of diabetes and cardiovascular disease) and diuretics (for those at high risk of heart failure); BB is the least favoured
The potential savings of using thiazides as first choice antihypertensive drug: Cost-minimization analysis (Fretheim et al. [23])	Thiazides, BB, CCB, ACEI, angiotensin II antagonists, alpha blocking agents	Survey-based estimates of the proportion of the adult population treated for hypertension in Canada, England, France, Norway, the US and Germany.	Canada, France, Germany, Norway, the UK and the US.	Cost-minimization analysis, thus assumes equal efficacy and tolerability	Favoured thiazide diuretics
Cost-effectiveness of Chlorthalidone, Amlodipine and Lisinopril as First-Step Treatment for Patients with Hypertension: An Analysis of the Antihypertensive and Lipid-Lowering Treatment to Prevent Heart Attack Trial (ALLHAT) (Heidenreich et al. [24])	Chlorthalidone (diuretic), lisinopril (ACEI), amlodipine (CCB)	Patients 55 years old or greater with hypertension and at least 1 additional risk factor for coronary heart disease	USA	Cost-effectiveness analysis	Favoured diuretics (chlorthalidone)
Economic evaluation of four angiotensin II receptor blockers in the treatment of hypertension (Miller et al. [25])	Olmesartan, losartan, valsartan and irbesartan	121,472 patients diagnosed with hypertension, aged 18 years or older; four cohorts based on drug used	Managed care setting, USA	Cost-effectiveness analysis with decision-analytic approach	Favoured olmesartan compared to the other ARBs
Evaluation of the awareness, control and cost-effectiveness of hypertension treatment in a Brazilian city: A populational study (Moreira et al. [26])	Diuretics, BB, ACEI, ACEI and diuretics, diuretics and BB, other medications	Sample representative of the adult urban population of Sao Paolo State, Brazil, 40 years or more; sample stratified by age groups and classified according to blood pressure level; study included 1492 individuals.	Brazil	Cost-effectiveness analysis in a randomized and cross-sectional populational study	Favoured diuretics
Analisis de costes farmacologicos en el tratamiento de la hipertension arterial: Aproximacion a un studio coste-efectividad (Moreno [27])	Comparison between ACE inhibitor, Beta-blocker and Calcium channel blocker	216 patients, 14 years or more; variables (age, sex, SBP, DBP, height, weight, heart rate) and risk factors (smoking history, obesity, hyperlipidemia and diabetes) taken	Primary care setting, Spain	Cost-effectiveness analysis	ACE inhibitor and Calcium channel blocker have greater cost-effectiveness ratio than diuretics. Diuretic was the least costly
Cost-Effectiveness analysis of treatments to reduce cholesterol levels, blood pressure and smoking for the prevention of coronary heart disease: Evaluative study carried out in Spain (Plans-Rubio [28])	Hydrochlorothiazide, nifedipine, propranolol, prazosin, captopril	Hypertension patients, mild, moderate severe based on DBP, 40–59 years old	Spain	Cost-effectiveness analysis	Favoured hydrochlorothiazide (diuretic), propranolol (BB) and nifedipine (CCB)
Analysis of Published Economic Evaluations of Angiotensin Receptor Blockers (Theodoratou et al. [29])	Olmesartan, telmisartan, candesartan, irbesartan, losartan and valsartan (all ARB)	Hypertension patients uncomplicated and with comorbidities; some populations had diabetes, albuminuria and nephropathy.	Studies used came from Japan, USA, UK, Spain, Canada, Switzerland, France, Belgium, Italy, Hungary, Germany, Sweden, Netherlands, Greece and South Africa. Two studies covered EU countries and Asia.	Literature review	No difference between available ARBs

ACEI – ACE inhibitor; ARB – angiotensin receptor blocker; BB – beta blocker; CCB – calcium channel blocker; DBP – diastolic blood pressure; HDL – high density lipid; LVH – left ventricular hypertrophy; SBP – systolic blood pressure.

All were economic studies on first-line hypertension treatment. Three were comparisons of drugs within the same class (ARBs). Nine studies compared 3 or more classes, including combinations of drugs and comparisons with no therapy. All studies aimed to identify the most cost effective drug/class for first-line treatment of hypertension. In general, health decision makers and policy makers were the target audience. National guidelines in particular had in mind prescribers because among their objectives was to assist in clinical decision-making.

Eleven studies classified the population sample according to hypertension or risk severity, while others classified them according to therapeutic class, response to therapy, age and sex. Patients came from different settings including primary care [22], managed care [25], a health facility [19] and at the sub-national level. Three studies came from middle-income countries (South Africa, Malaysia and Brazil) while eight came from high-income countries. The three literature reviews generated studies from several high-income countries. The studies had different funding sources. Those funded by the industry tended to favour their particular molecule while the studies funded by governments tended to have cost savings in mind.

### Technical characteristics

Five studies were done using the health care perspective while four studies were performed using the third party payer/ social insurance perspective. Time horizons varied according to the design and objectives of the study. The methodology used were economic analyses namely cost-minimization (CMA), cost-effectiveness (CEA) and cost-utility analyses (CUA). Theodoratou’s systematic literature review [29] had similar methodology to this study. The standard definition of hypertension was used except in two studies where only systolic (blood pressure) BP [20] and diastolic BP [28] were used. These differences do not raise technical questions since it is the cost and effectiveness that are being compared using comparable blood pressure levels.

Parameters (probabilities and risks) were obtained through literature review, existing studies and trials, local clinical data or databases or through risk equations. Ten (10) studies included direct costs and none included indirect costs which is still a standard recommendation. Effectiveness measures were varied according to the type of economic study. Among the cost-effectiveness and cost-minimization analyses, four studies used BP reduction while the other studies used proportion of patients with controlled BP, long-term outcomes (e.g. strokes or MIs prevented) and life years gained. Both cost-utility studies used quality adjusted life-years (QALYs) along with life years gained. Only the two cost-utility analyses used discounting at 5% (Canada) and 3.5% (UK) for base cases. Four studies mentioned assumptions regarding drug effects while the rest did not mention assumptions.

Results were presented as cost-effectiveness ratios (CER), incremental cost-effectiveness ratios (ICER) or both implying that some studies were looking at average cost-effectiveness rather than incremental cost-effectiveness. As expected, the two cost-minimization analyses mentioned costs or incremental costs. Eight studies performed sensitivity analysis.

Generalizability issues were mainly considerations on the country sources of parameters and the age range. The findings of the Malaysian study [19] may be relevant to other developing Southeast Asian countries, considering that the proportions (of controlled hypertensive patients) were based on a local database. Limitations discussed were mostly technical concerns. These were related to the drugs/ therapeutic classes included; sampling, inclusion and randomization of patients (if applicable); population/cohort characteristics; sources of parameters and data; and the measure of effectiveness data. Incorporating clinical considerations in the models would increase their complexity. Hence, non-inclusion of adverse events, switching of therapy, combination therapy and withdrawal of therapy is always a limitation. In addition, data on their relative risks may not have been available.

The authors’ conclusions of cost-effectiveness were based on the criteria that they were using. Some referred to WHO’s cost-effectiveness cut-off of CER within 3 times the country’s GDP, while some based their conclusion on their country’s willingness to pay. Eight studies concluded in favour of diuretics versus other classes. NICE [22] recommended both diuretics and CCB among patients more than 55 years and ACEI/ARB among patients below 55 years. In general, the clinical efficacy of different drugs did not vary greatly but diuretics were favoured mainly because of their low cost. NICE reiterated that their results were sensitive to the cost of CCBs and the risk profile of patient groups. For patients with high risk of heart failure and diabetes, ACEIs or ARBs were the best choices. CCBs were associated with low risk of diabetes and had a good profile across the range of other CVD risks. In addition, the cost-effectiveness results were dependent on the society’s willingness to pay.

Theodoratou [29] concluded in favour of ARBs versus other classes when co-morbidities are considered. Theodoratou showed no significant difference among drugs within this class while other ARB studies concluded in favour of Candesartan [20] and Olmesartan [25]. None of the studies included a discussion of socio-political implications and feasibility. Several recommendations were discussed 1) for decision-making and treatment options, 2) for enhancement of the economic evaluation and 3) for strategies to reduce drug costs.

## Discussion and policy implications

### Cost-effective modalities

The twelve reviewed articles were able to compare all anti-hypertension drug classes using different economic methods and in different settings/countries covering both the health and societal perspectives. The target audiences included decision-makers and practitioners. Hence, the selected studies had a good overall representation and the conclusions may be applied to the Philippine context and other middle-income settings.

The articles that compared different classes of drugs were agreed in recommending diuretics as the most cost-effective drug class for first-line treatment of hypertension without co-morbidities. They recognized that the clinical efficacy of different classes in lowering hypertension were comparable. It is certainly the low cost of diuretics that puts them at an advantage [24,30,31]. This finding is consistent with clinical guidelines which are mostly based on clinical trials. The use of diuretics is particularly relevant in developing countries where resources are limited [32].

Identifying the cost-effective drug classes for hypertension patients with co-morbidities will require another set of economic evaluations. This will be a larger task since evaluations must be according to subgroups, i.e. patients with history of diabetes, stroke, myocardial infarction, congestive heart failure, renal failure, and so on [7]. From a public health perspective, economic studies on hypertension management go beyond medical treatment and include preventive strategies, education, diagnostic procedures, non-medical interventions and comprehensive management. Recent studies have shown the cost-effectiveness of lifestyle modification, population based interventions, tobacco regulation and comprehensive community-based hypertension programmes that adhere to clinical guidelines [28,32-37]. A risk-approach to treatment is also more cost-effective than the usual approach that is based on BP levels only [38]. Treatment of moderate to high risk patients is more cost-effective than treatment of low risk patients [7,32]. Recent guidelines even recommended no or minimal treatment of low risk patients [39,40].

### Diuretics as first-line treatment in the Philippines

Although diuretics have been recommended in the Philippine Clinical Guidelines and in the JNC 7 (The Seventh Report of the Joint National Committee on Prevention, Detection, Evaluation, and Treatment of High Blood Pressure; guidelines followed by most specialists in the country), diuretic utilization is very low (in fact nil) as seen in PhilHealth in-patient claims. It can be argued that in-patient cases are more advanced. Interestingly, the beta-blocker metoprolol is still the most popular drug (even in hospitalized patients). In fact, the new programme P100 that is being piloted in a few areas subsidizes two beta-blockers and two calcium channel blockers (in addition to diuretics) that are prescribed on an out-patient basis [41]. Considerations on efficacy, safety, availability, access and costs and pharmaceutical advertising have been identified as possible reasons [42,43]. It is difficult to conclude however because there is no data showing utilization of anti-hypertensive medicines at the community level where most low-risk cases might be found and the reasons why other drug classes are preferred are unknown. There is also no published analysis on how current medicines policies (or lack of these) govern prescribing practices.

Previous studies have shown that adherence to treatment guidelines, assuming that these guidelines have cost-effectiveness as a value, would result to savings in the health care delivery system. On the other hand, even the presence of economic data does not ensure that the third party payer and public health sector can effectively implement its recommendations. Promoting the use of clinical guidelines however will require initial cost but the long term savings are projected to be greater [30,38].

Following recommendations of previous studies, PhilHealth may recommend and subsidize diuretics as first-line treatment on an out-patient basis. Since economic evidence has shown future savings from health care costs due to complications, covering for out-patient diuretics may be a good investment. Depending on society’s willingness to pay, calcium channel blockers and ACE inhibitors may be subsidized as well. The studies conducted at the sub-national level, primary care setting and health facility showed the cost-effectiveness of diuretics. Elsewhere it was described that comprehensive strategies for hypertension were cost-effective at the sub-national level [28,36]. Generalizing these recommendations for application at a Philippine local setting is predicted to produce savings.

Having answered which medicines are cost-effective by reviewing the literature leads to a new set of economic questions at the national policy level. Is publicly funded first-line anti-hypertensive treatment justified in economic terms? This question is raised considering that the government or PhilHealth does not necessarily subsidize long-term care. Subsidizing out-patient medicines for hypertension in order to prolong their productive years assumes that majority of such patients are economically productive (which leads to the question of what is economically productive). National decisions to cover first-line anti-hypertensive treatment should also consider competing interests to prevent cardiovascular diseases including education, advocacy, lifestyle modification, healthy diet, physical activity, restriction of access to alcohol and tobacco, etc. When hypertension control and management competes with other health priorities, to which should funds be allocated? Answering these questions will require a strong economic evaluative capacity for any country. WHO-recommended best-buys for NCDs and the support of the United Nations General Assembly to control NCDs are important milestones, but middle-income countries should also exercise caution when deciding where to allocate funds.

### Application of economic evaluation for decision making in the Philippines and other middle-income countries

Economic evaluation is a major component of health technology assessment and is a key element in evidence-based policy-making. Informed decisions are able to select the best mixture of cost-effective options that will promote allocative efficiency and will increase value for money within limited resources [44]. Economic evaluation will certainly benefit PhilHealth and the DOH in decision-making in terms of resource allocation and will assist guideline development and provide evidence to support PhilHealth reimbursement/coverage including out-patient benefit packages.

Economic evaluation has different roles in different country-systems. A mixed health care system, i.e. a public sector with a strong market-based sector, is seen in many growing economies such as India, Brazil, Indonesia and the Philippines. In such settings economic evaluation may also be used to: 1) evaluate payment schemes for and budgets of health care institutions, 2) plan payment systems for health care providers, 3) evaluate cost-sharing arrangements, 4) encourage competition and 5) review utilization of medical technology [14].

Economic evaluation is feasible in the Philippines; the methodology and variables will depend a lot on the policy questions and objectives. This review of economic literature is already relevant to and may be applied in the Philippine context. In cost-minimization analysis, the effectiveness of the interventions in comparison is assumed to be equal. Sources of information on costs can be acquired from health programme expenses, health care billings and drug prices. Other than hypertension costs from PhilHealth claims and reimbursements [3], data on out-of-pocket and local government expenditures are still lacking. Thus, there are already information gaps in terms of how much health care costs in the Philippines. A survey of general health care costs covering different sectors – PhilHealth, DOH, local government, private sector, pharmacies and private individuals – not just on cardiovascular diseases but on major disease entities will be very beneficial for health decision making. An investment on this research has to be made.

Cost-effectiveness analysis requires effectiveness measures which can either be intermediate outcomes (e.g. BP control or proportion of patients with controlled BP) or long-term outcomes (e.g. life years gained, MI/stroke averted). This information may be obtained through review of published clinical data although most of these would be coming from high-income countries. Local clinical data will give different values because of the differences in lifestyles, prevention strategies and health care delivery systems. These may be obtained through health facilities and programmes in different geographic locations (as was done in Brazil and Malaysia) but will require funding and research.

Cost-utility studies require values on transition probabilities, relative risks and risk reductions which can be derived from trials done in high-income countries. On the assumption that disease history and responses to treatment are uniform worldwide, there should not be much difference in these parameters. Risk equations such as the Framingham equation may also be used to derive these values. On the other hand, if lifestyles and prevention measures are taken into account, these parameters may differ in developing countries. For instance, salt and oil/fat content in food are not regulated in the Philippines. Thus, locally derived probabilities, relative risks and risk reductions may produce slightly different values. Utilities in the two cost-utility analyses included in the study [5,16] were derived from North American and European populations. It has not been shown whether utility values will differ in middle-income countries because of the differences in lifestyle, preventive measures, access to health care, health care delivery system, social values, illness experiences and perceptions of ill health. Thus, it may be advisable to obtain data on QOL values in developing countries. There is a paucity of studies showing differences of QOL values in low-, middle- and high income countries.

Comprehensive and community-based health programmes and projects are being done in many parts of the world including in the Philippines. These activities are initiated by various stakeholders including the national government, local governments, international agencies, the private sector and NGOs. These programmes include both communicable and non-communicable diseases along with other public health initiatives (e.g. nutrition and injury prevention and control). These initiatives are good opportunities for costing and economic analyses depending on the relevant questions. The non-use and non-awareness of the population and stakeholders on economic evaluation and evidence-based policies is a reality in developing countries. Lack of policy direction, resources and expertise are among the limiting factors. The challenge is for the policy-makers, stakeholders, academia, health advocates and community mobilizers to know the value of these tools. There is beginning interest, since many middle-income countries are setting up HTA agencies, but technical capacity will be a limiting factor.

Economic evaluation as a governance/policy tool supports the priorities of the Philippine government to enhance health financing and access to medicines by providing information to improve efficiency. Considering that health financing, health technology and health information are among the building blocks of a health system, it is essential that policies, expertise and structures for economic evaluation within health technology assessment are strengthened. The DOH, PhilHealth and local government units must incorporate economic aspects in planning, research and decision making if relevant. International agencies (including the WHO, the World Bank and other UN agencies) and NGOs may also include economic evaluation in proposals, planning and evaluation. On the other hand, it is a challenge for health economists and trained researchers/policy-makers to enhance the awareness of decision-makers, public officials, the media, the academe, the industry, health insurance sector, health professionals, patient/consumer organizations and the general public with regard to the benefits of economic evaluation [12].

## Conclusions

In this study, I reviewed 12 economic studies for treatment of hypertension that represented different countries, perspectives and stakeholders. Diuretics were the most cost-effective drug class for first-line treatment of hypertension without co-morbidities. The clinical efficacy of different classes in lowering blood pressure is comparable and the advantage of diuretics is their low cost. In spite of its strong recommendations from guidelines and clinical trials, diuretic utilization among hospitalized patients in the Philippines is low. Clinical guidelines developed with cost-effectiveness in mind promote future savings in the health care management of complications. Although PhilHealth may apply the recommendations given in previous studies (i.e. to subsidize diuretics, ACE inhibitors and calcium channel blockers), it is uncertain how much public funding is justified.

Middle-income countries such as the Philippines should consider economic evaluation as a component of health technology assessment. The objectives and questions will be context-specific. Data on costs can be acquired locally. Risk reductions, transition probabilities, relative risks and utility values may be acquired from international studies although local data would be more relevant. There seems to be an information gap on clinical data and utility values on hypertension and related diseases from middle- and low- income countries. This study is only a review of existing literature. The conduct of actual field survey or data gathering will depend on the kind of economic question.

If the Philippine government desires to subsidize first-line hypertensive medicines, it should do so for cost-effective drugs namely diuretics, calcium channel blockers and ACE inhibitors. PhilHealth and the Department of Health should work with the private and local government health sectors to promote the use of these medications according to the Philippine guidelines for hypertension. Local governments that provide anti-hypertensive medications for their constituents must also follow these recommendations. Management of hypertension patients must take a comprehensive and primary care approach. A study on the costs of hypertension including inpatient, outpatient, out-of-pocket, local government and national government expenditure must be made.

Economic evaluation may be incorporated in health technology assessment, planning, research and evaluation of health programmes. The approaches will vary depending on the policy questions. Government agencies, international agencies, including WHO and the World Bank, and NGOs in the Philippines may benefit from economic evaluation. Awareness of the value of economic evaluation must be strengthened among health and policy makers, professionals and the general public. Strengthening economic evaluative capacity is a key component as growing economies readjust their resources to meet increasing health and systems concerns.

## Abbreviations

ACEIs: Angiotensin converting enzyme (ACE inhibitors; ALLHAT: Antihypertensive and lipid-lowering treatment to prevent heart attack trial; ARBs: Angiotensin II receptor blockers; BBs: Beta blockers; BP: Blood pressure; CCBs: Calcium channel blockers; CEA: Cost-effectiveness analysis; CER: Cost-effectiveness ratio; CMA: Cost-minimization analysis; CUA: Cost-utilization analysis; DOH: Philippines department of health; FDA: Philippines food and drugs administration; HTA: Health technology assessment; ICER: Incremental cost-effectiveness ratio; JNC VII: The seventh report of the joint national committee on prevention, detection, evaluation, and treatment of high blood Pressure; NGO: Non-government organization; NHS: UK national health service; NICE: UK national institute for health and clinical excellence; PhilHealth: Philippine health insurance corporation; WHO: World health organization.

## Competing interests

My MSc(2010–2011) for which this study was conducted was funded through the Joint Japan World Bank Graduate Scholarship Programme (JJWBGSP). There are no other competing interests.

## Authors’ contribution

LSAG conducted all methodology, drafting and finalization for this study as a dissertation requirement for MSc – Health Policy, Planning and Financing at the London School of Economics and Political Science (LSE) and the London School of Hygiene and Tropical Medicine (LSHTM).

## Authors’ information

LSAG is a graduate school student at the London School of Economics and Political Science (LSE) and the London School of Hygiene and Tropical Medicine (LSHTM).
